# Excessive excitability of inhibitory cortical circuit and disturbance of ballistic targeting movement in degenerative cerebellar ataxia

**DOI:** 10.1038/s41598-023-41088-3

**Published:** 2023-08-25

**Authors:** Akiyoshi Matsugi, Satoru Nishishita, Kyota Bando, Yutaka Kikuchi, Keigo Tsujimoto, Yuto Tanabe, Naoki Yoshida, Hiroaki Tanaka, Shinya Douchi, Takeru Honda, Masato Odagaki, Hideki Nakano, Yohei Okada, Nobuhiko Mori, Koichi Hosomi

**Affiliations:** 1 Faculty of Rehabilitation, Shijonawate Gakuen University, Hojo 5-11-10, Daitou City, Osaka 574-0011 Japan; 2 Institute of Rehabilitation Science, Tokuyukai Medical Corporation, 3-11-1 Sakuranocho, Toyonaka City, Osaka 560-0054 Japan; 3 Kansai Rehabilitation Hospital, 3-11-1 Sakuranocho, Toyonaka City, Osaka 560-0054 Japan; 4 National Center Hospital, National Center of Neurology and Psychiatry, Kodaira, 187-0031 Japan; 5 Department of Rehabilitation for Intractable Neurological Disorders, Institute of Brain and Blood Vessels Mihara Memorial Hospital, Ohtamachi 366, Isesaki City, Gunma 372-0006 Japan; 6 Okayama Healthcare Professional University, 3-2-18 Daiku, Kita-ku, Okayama City, Okayama 700-0913 Japan; 7 KMU Day-Care Center Hirakata, Kansai Medical University Hospital, Shinmachi 2-3-1, Hirakata City, Osaka 573-1191 Japan; 8 Department of Physical Medicine and Rehabilitation, Kansai Medical University, Shinmachi 2-5-1, Hirakata City, Osaka 573-1010 Japan; 9 Department of Rehabilitation, National Hospital Organization Wakayama Hospital, Hukakusamukaihatacyo1-1, Husimi-ku, Kyoto City, Kyoto 612-8555 Japan; 10 The Center for Personalized Medicine for Healthy Aging, Tokyo Medical and Dental University, 1-5-45 Yushima, Bunkyo-ku, Tokyo, 113-8510 Japan; 11 Maebashi Institute of Technology, Maebashi, Gunma Prefecture Japan; 12 Department of Physical Therapy, Faculty of Health Sciences, Kyoto Tachibana University, Kyoto, Japan; 13 Neurorehabilitation Research Center of Kio University, Koryo-cho, Kitakatsuragi-gun, Nara 635-0832 Japan; 14 Department of Neurosurgery, Osaka University Graduate School of Medicine, Yamadaoka 2-2, Suita City, Osaka 565-0871 Japan

**Keywords:** Cerebellum, Motor cortex, Movement disorders, Neurodegeneration, Spinocerebellar ataxia

## Abstract

This study aimed to investigate abnormalities in inhibitory cortical excitability and motor control during ballistic-targeting movements in individuals with degenerative cerebellar ataxia (DCA). Sixteen participants took part in the study (DCA group [n = 8] and healthy group [n = 8]). The resting motor-threshold and cortical silent period (cSP) were measured in the right-hand muscle using transcranial magnetic stimulation over the left primary motor cortex. Moreover, the performance of the ballistic-targeting task with right wrist movements was measured. The Scale for the Assessment and Rating of Ataxia was used to evaluate the severity of ataxia. The results indicated that the cSP was significantly longer in participants with DCA compared to that in healthy controls. However, there was no correlation between cSP and severity of ataxia. Furthermore, cSP was linked to the ballistic-targeting task performance in healthy participants but not in participants with DCA. These findings suggest that there is excessive activity in the gamma-aminobutyric acid-mediated cortical inhibitory circuit in individuals with DCA. However, this increase in inhibitory activity not only fails to contribute to the control of ballistic-targeting movement but also shows no correlation with the severity of ataxia. These imply that increased excitability in inhibitory cortical circuits in the DCA may not contribute the motor control as much as it does in healthy older adults under limitations associated with a small sample size. The study's results contribute to our understanding of motor control abnormalities in people with DCA and provide potential evidence for further research in this area.

## Introduction

Degenerative cerebellar ataxia (DCA)^1^ is an inherited neurodegenerative disorder characterized by progressive cerebellar ataxia^2^. Representative diseases include spinocerebellar ataxia (SCA)^3–5^, Friedreich's ataxia^6^, the cerebellar subtype of multiple system atrophy (MSA-C)^7,8^, autosomal dominant spinocerebellar ataxia (ADSCD)^9^, and sporadic adult-onset ataxia of unknown etiology (SAOA)^10^. The pathological hallmark of nearly all forms of DCA is the loss of neurons, particularly in the Purkinje cell layer of the cerebellum, resulting in cerebellar atrophy and the subsequent onset of cerebellar ataxia^11^. Additionally, alterations in the function of other areas of the brain, such as the primary motor cortex (M1), have been observed in individuals with DCA^12–15^. Corticospinal excitability, which is estimated based on the motor-evoked potential (MEP) induced by single-pulse transcranial magnetic stimulation (TMS) to M1, tended to decrease. The excitability of the gamma-aminobutyric acid (GABA)-mediated inhibitory circuit in M1^16,17^, which is estimated based on the cortical silent period (cSP) accompanied by MEP, is abnormally prolonged^18–20^. However, it remains unclear whether these neurophysiological parameters obtained from intact M1 tissue are associated with DCA disease-specific movement disorders.

cSP reflects the GABA-mediated excitability of inhibitory neural circuits in the M1^16,17^. The cSP is modulated depending on muscle contraction to control forces such as finger movement^21^. Healthy subjects with longer cSP have been reported to perform better on motor cancellation tasks^22^. Furthermore, cSP is modulated by conditioning with cerebellar single-pulse TMS, indicating that the cerebellum has an output to the GABA-mediated inhibitory circuit in M1^23^. Individuals with DCA have impaired force control^24^. Therefore, cSP may be associated with motor control dysfunction in patients with DCA.

The Scale for the Assessment and Rating of Ataxia (SARA) is commonly used as an indicator of the severity of ataxia in individuals with DCA^25^. Therefore, we examined the relationships between these parameters and SARA. However, SARA roughly grades the degree of motor impairment and does not express specific abnormalities such as prolongation of movement time^26^, degree of dysmetria^27^, or degree of delayed motor onset due to a distorted internal timer^28^. To represent these motor impairments specifically, observing behavior during a specific task movement is necessary. Therefore, we devised a ballistic targeting task that included reacting to the cue to start a movement as fast as possible to move and stop at the target angle of the joint and examined the contribution of the cerebellum^29^. After low-frequency cerebellar repetitive TMS, the reaction time (RT), maximum velocity (MV), movement time (MT), and targeting error (TargetE) were significantly changed, indicating that this ballistic targeting task was associated with cerebellar function. Based on these findings, we hypothesized that the function of the inhibitory circuit in the M1 for movement is associated with ballistic-targeting tasks and cSP may correlate with performance on this ballistic-targeting task.

This study examined whether motor cortex function associated with GABAergic inhibitory circuits is an indicator of ataxia and motor performance in patients with DCA. More specifically, we examined whether cSP and resting motor threshold (rMT) were related to SARA and time-, speed-, and accuracy-related parameters of ballistic targeting movements. Given that cSP is specifically prolonged in patients with DCA^18–20^, a positive correlation between cSP and SARA in DCA and a negative correlation between SARA and these measures of exercise performance are expected.

## Materials and methods

### Participants

Sixteen adults (people with DCA, n = 8; age-matched healthy adults, n = 8) participated in this study. They were diagnosed with ADSCD^9^, SAOA^10^, multiple system atrophy (MSA), MSA-C^7^, or spinocerebellar ataxia type6 (SCA6)^5^ or type31(SCA31)^4^. The DCA group had a mean (± standard deviation) of 107.3 ± 64.0 months since diagnosis and a total SARA (mean ± standard deviation) score of 18.8 ± 5.9. The DCA and age-matched healthy groups had a mean (± standard deviation) age of 63.4 ± 11.2 and 63.3 ± 11.1 years, respectively. The number of male and female individuals in the DCA and healthy control groups was 4 and 4, respectively. In the healthy control group, all the participants declared that they had no history of neurological disorders.

This study was approved by the Ethics Committees of Shijonawate Gakuen University (Approval Code: 20-4), National Center of Neurology and Psychiatry (Approval Code: A2020-113), and Institute of Brain and Blood Vessels Mihara Memorial Hospital (Approval code: 104-03). All participants were briefed about the experiment and signed a consent form before participation. The study was conducted in accordance with the principles and guidelines of the Declaration of Helsinki.

### General procedure

The rMT, cSP, and motor performance in ballistic targeting tasks in patients with DCA were tested at the National Center of Neurology and Psychiatry in National Center Hospital (NCNP) and Institute of Brain and Blood Vessels Mihara Memorial Hospital (MMH), and those in healthy subjects were tested at Shijonawate Gakuen University. All tests were conducted on the same day. SARA in patients with DCA was measured by a skilled physical therapist at NCNP and MMH. The period (months) since diagnosis was obtained from the medical records of each hospital. However, this was not the period from the disease onset. An age-matched healthy control group was recruited after enrolling all the patients with DCA.

### cSP measurement

**Figure 1:**
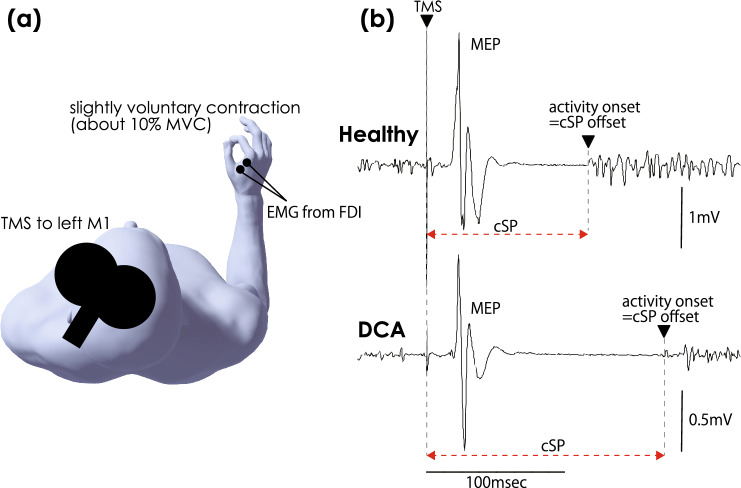
Setting for cSP measuring and typical waveform. (**a**) The setting for measuring the cortical silent period (cSP). The participant was seated, and electromyography (EMG) was recorded from the right first dorsal interosseous muscle (FDI). The TMS coil was set on a hotspot for inducing motor-evoked potential (MEP) on FDI-EMG of the left primary motor cortex (M1). (**b**) The typical waveform of EMG in healthy participants (top) and individuals with degenerative cerebellar ataxia (DCA) (bottom). The solid triangle indicates the timing of TMS, and motor-evoked potential (MEP) appeared with approximately 20-msec latency from TMS. The cSP was detected following MEP, and reactivation appeared.

Figure 1 illustrates the settings for the rMT and cSP measurements. The participants were seated on a chair. To record the electromyography (EMG) signals, two Ag/AgCl surface-recording electrodes were placed 2 cm apart on the right first dorsal interosseous (FDI) muscle, and a reference electrode was placed on the right dorsal wrist. The EMG signals were amplified using an amplifier (MEG-1200; Nihon Kohden, Tokyo, Japan) with a bandpass filter of 15 Hz to 3 kHz. The EMG signals were converted into digital signals at a sampling rate of 10 kHz using an A/D converter (PowerLab 800S; AD Instruments, Colorado Springs, CO, USA), and the digital signals were stored on a personal computer. The recording method was identical to that reported in our previous study^21^.

A TMS pulse was delivered to M1 using a figure-of-8 coil (D70; The Magstim Company Ltd., Spring Gardens, UK) connected to a magnetic stimulator (The Magstim; The Magstim Company Ltd.). The center of the junction of the figure-of-8 coil was set at the hotspot on the left M1 to induce MEPs in the right FDI. A hotspot was defined as the site where the largest MEP was obtained in the left hemisphere of the head of the participant. This hotspot was marked on the swimming cap that the participants wore to prevent changes to the stimulation site. The current in the coil was directed from posterior to anterior, inducing an AP traveling current in the brain^30,31^. The rMT of the FDI muscle was defined as the minimal intensity of the magnetic stimulator output that produced MEPs with an amplitude larger than 50 µV in at least three out of five stimulations delivered over the hotspot^21,32,33^. To measure the cSP, the stimulus intensity was set at 1.3 × the rMT for measuring cSP. A TMS pulse was delivered during a slight contraction of the right FDI. Thirty pulses were applied at intervals longer than 7 sec.

### Ballistic targeting motor task

**Figure 2:**
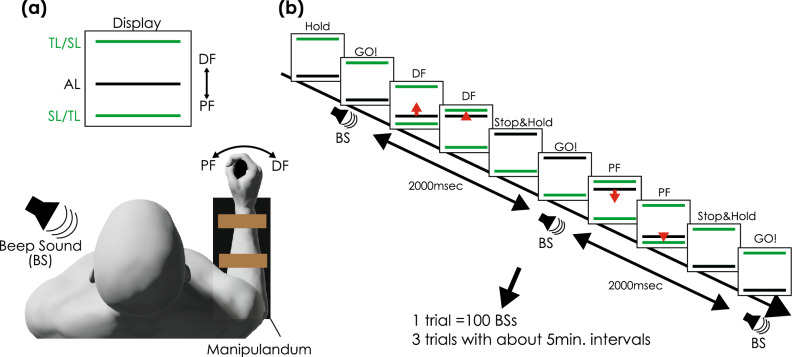
Setting and procedure for ballistic targeting task. (**a**) The participant sat on the chair in front of the display. The right forearm was fixed on the manipulandum, and the participant gripped the handle. The angle line (red line) moves upward with dorsiflexion (DF) and downward with plantarflexion (PF). (**b**) First, the angle line was set on the bottom target line (green line). Next, the participant moved the angle line with DF as fast as possible to the top target line immediately after the beep sound. The participant held the position of the angle line on the top target line until the next beep sound. When the next beep was sounded, the angle line was moved as fast as possible to the bottom target. The interval of beep sound was set to 2000 msec, and 100 beep sounds were applied during trial 1, indicating that trial 1 included 50 DF and 50 PF. Three trials were conducted with approximately 5-min intervals for rest.

Figure 2 illustrates the settings and procedure of the ballistic-targeting motor tasks. Each participant was seated on a chair that was 1 m in front of the monitor. A handmade manipulandum was used in the previous study^29^. The handling movement was designed to prevent resistance. The angle signals were recorded at a sampling rate of 1 kHz using an A/D converter (PowerLab 800S; AD Instruments) on a personal computer. The participants were instructed to move the angle line on the display using a handle (Fig. 2). The angle line moved upward with dorsiflexion (DF), and downward with plantarflexion (PF). The target line was set at approximately 35° to the DF and PF, and to ensure comfortable motion, slight adjustments were made depending on the size of each hand.

Initially, the participants set an angular line on the bottom line. During the first beep, the participants moved the angle line to the target line (upper line) as quickly as possible and maintained the angle line on the target line (upper line) with the DF of the wrist. When the next beep sounded, they quickly moved the angle line to the next target line (bottom line) from the upper line using the PF of the wrist and maintained it there. One hundred beep sounds were applied at 2000-msec intervals in one trial. Therefore, the targeting tasks in the DF and PF directions were repeated 50 times for one trial. Three trials were conducted, with approximately five-minute intervals for rest.

### Analysis

Figure 1 shows the specimen records of EMG from the FDI muscle in patients with DCA and healthy controls. The vertical line indicates the TMS artifacts. The MEP was described on the right side of the TMS artifact with approximately 20 msec latency. An EMG burst can be detected after a silent period. cSP duration was defined as the interval between TMS and the onset of the EMG burst^16,21,34^. The offset of the cSP was defined as > mean + 3 × SD of the bEMG level in the resting state^21,34^; however, if the EMG baseline drifted, the offset was visually estimated. Therefore, two independent assessors measured the cSP, and the average of the two cSP values was used for subsequent analyses. The independent assessors were selected based on their experience in the previous analysis^23^.

**Figure 3:**
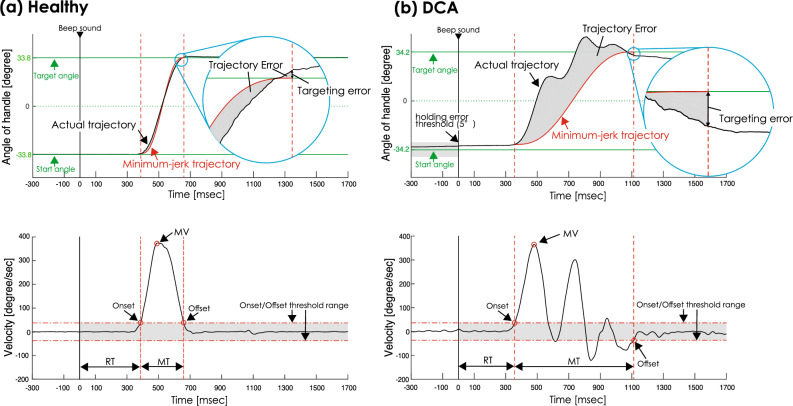
Typical waveform of angle and velocity in (**a**) Healthy and (**b**) degenerative cerebellar ataxia (DCA) groups. The tops indicate the waveform of the angle of the handle, and the bottom indicates the velocity waveform. The horizontal scales indicate time. The vertical scales indicate the angle (degree) of the handle in the top waveforms and the velocity (degree/sec) of handle movement in the bottom waveforms. The green lines indicate the star/target angle. The black lines indicate the actual trajectory in top waveforms and the calculated velocity in bottom waveforms. The vertical red dot lines indicate onset and offset timing. The difference between the actual angle and the target angle is defined as a targeting error in top waveforms. The red lines indicate minimum-jerk trajectory in the top waveform. The gray area between actual and minimum-jerk trajectory is trajectory error. Abbreviations: RT, reaction time; MV, maximum velocity; MT, movement time.

Figure 3 shows the specimen waveforms of the handle angle in the Healthy and DCA groups during the ballistic targeting task. First, to obtain the summation indicator of the performance in this ballistic targeting movement task for each individual, the angle data for the PF were multiplied by − 1, which allowed it to be treated as a DF assignment. In the PF direction task, the handle angle decreased when the target line moved from the top to the bottom. Next, the angle (degrees) was converted to velocity (degrees/sec) (Fig. 3, bottom line), and the maximum velocity (MV) was detected. We excluded any trials with more than 1000 degrees/s of MV by visually checking the waveform. If a clear artifact or signal noise was visually identified, the trial was excluded from the analysis. This is because velocity cannot occur in normal motion and does not capture the essence of motion. If there was a deviation of more than 5° from the start line at the time of the beep sound, the participant was considered to have a "holding error," and if movement occurred at a speed of more than 5 degrees/300 msec before beep sound, the participant was considered to have a "false start.” This was used as an indicator of failure to perform the task due to abnormal muscle tone or tremor caused by ataxia or inability to measure the timing of the movement due to timer distortion.

Movement onset and offset were defined as the time at over and under 10% MV, respectively. RT was defined as the time from the beep sound to movement onset (Fig. 3). Movement time (MT) was defined as the time between movement onset and offset. The absolute targeting error (TargetE) was defined as the difference between the target and actual handle angles at the time of movement offset. To estimate the tremor and decomposition of the trajectory, the trajectory error (TrajectE) was calculated as the integral absolute value of the difference between the actual trajectory and minimum-jerk trajectory of the angle. If dysmetria is present, TargetE will increase. The minimum-jerk trajectory^35^ was calculated as the trajectory that minimizes the time-integrated value of the square of the jerk angle at the MT. If a perfectly efficient exercise with a unimodal profile of velocity peaks is performed, the value of TrajectE will be zero; however, if an exercise involving motion decomposition that results in a velocity profile with two or more peaks is performed, TrajectE will be large. If the performance in the ballistic targeting task is high, RT, MT, TrajectE, and TargetE will be small, and MV will be high.

To test for differences in age, cSP, and rMT, a t-test was conducted based on the results of Levene’s test for equality of variances between the Healthy and DCA groups. If variances were not equal, a non-parametric test (Mann–Whitney U test) was used instead of the t-test. Receiver operating characteristic (ROC) curve analysis was performed to estimate the ability of cSP to differentiate between healthy individuals and individuals with DCA. To compare the rate of premature task errors, including "holding error" and “false start” between the healthy and DCA groups, the Chi-square test was conducted.

To estimate the effect of disease (Healthy or DCA) and the number of trials (Trials 1, 2, and 3) on task performance (RT, MV, MT, TrajectE, and TargetE), a two-way analysis of variance (ANOVA) was conducted. However, if equality of variances was not confirmed by Levene’s test, a non-parametric test (Kruskal–Wallis test) was used to estimate the effect on these parameters. If any factor affected the parametric or non-parametric ANOVA, we conducted a post-hoc test (t-test or Mann–Whitney U test) to compare healthy individuals to patients with DCA for each trial. We also performed multiple comparisons between trials 1, 2, and 3 within disease groups, with Bonferroni correction applied (alpha level, 2 inter-disease, 3 inter-trial).

To investigate whether there was a correlation between cSP, SARA, and exercise performance measures, we conducted separate correlation analyses for the DCA and healthy control groups. The Shapiro–Wilk test was conducted to test for normality. If the Shapiro–Wilk test indicated a non-normal distribution of data, Spearman’s test was applied. If the distribution was normal, Pearson’s test was applied. All statistical analyses were conducted using JASP software (version 0.17.1; University of Amsterdam, Amsterdam, Netherlands)^36^, and the alpha level was set at 0.05.

### Ethics approval

This study was approved by the Ethics Committees of Shijonawate Gakuen University (approval code: 20-4, 19-9), National Center of Neurology and Psychiatry (approval code: A2020-113), and Institute of Brain and Blood Vessels Mihara Memorial Hospital (approval code: 104-03).

### Informed consent

All participants were briefed on the experiment and signed a consent form before participation.

## Results

Data were collected from 16 participants. There were no side effects of single-pulse TMS or ballistic targeting tasks.

### Inhibitory cortical excitability

**Figure 4:**
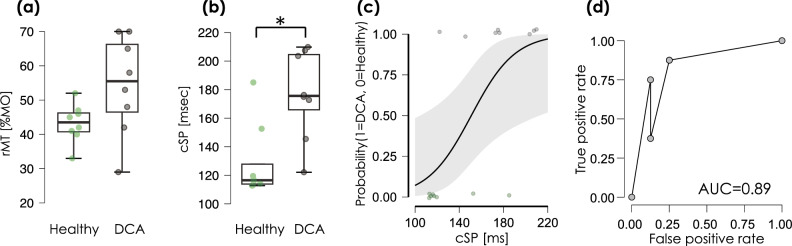
rMT and cSP. (**a**) The resting motor threshold (rMT) and (**b**, **c**) cortical silent period (cSP) in Healthy and degenerative cerebellar ataxia (DCA) groups. The green jitter elements indicate the individual data of Healthy, and the black jitter elements indicate DCA. The asterisk indicates a significant difference. (**d**) The receiver operating characteristic curve (ROC) for detecting the function of cSP to distinguish DCA from Healthy. The area under the curve (AUC) is 0.89. %MO; percent of maximum output intensity of magnetic stimulator.

Levene’s test revealed equal variance in age (F = 0.002, *p* = 0.964) and cSP (F = 0.102, *p* = 0.754) but not in rMT (F = 5.711, *p* = 0.031) between the Healthy and DCA groups. The t-test or Mann–Whitney test revealed that there was no significant difference in age (t = 0.022, df = 14, *p* = 0.982) and rMT (U = 13.5, df = 14, *p* = 0.058) between Healthy and DCA (Fig. 4-a,); however, there were significant differences in cSP (t = -3.324, df = 14, *p* = 0.005) (Fig. 4-b, c) between the Healthy and DCA groups. Figure 4-d indicates the ROC for the indicator of the ability to detect DCA from the healthy population. Logistic regression analysis revealed that cSP had significant discriminative power (Akaike's Information Criterion = 18.046, Bayesian Information Criterion = 19.591, df = 14, chi-square = 8.135, *p* = 0.004, McFadden R^2^ = 0.367), and the area under the curve (AUC) was 0.891.

### Motor control findings

Trials that could not be analyzed due to obvious artifacts were detected in 15/2400 and 0/2400 in the DCA and Healthy groups, respectively. The chi-square test revealed that the rate of premature task error, including holding error and a false start, in the DCA group (135/2385) was significantly higher than that in the healthy group (20/2400) (n = 4785, chi-square = 88.927, *p* < 0.001).

**Figure 5:**
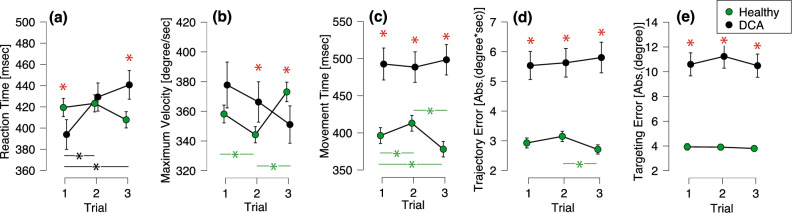
Performance of ballistic targeting task: (**a**) reaction time, (**b**) maximum velocity, (**c**) movement time, (**d**) trajectory error, and targeting error. The green circles indicate the healthy group, and the black circles indicate the degenerative cerebellar ataxia (DCA) group. The green asterisks indicate the significance between trials in the healthy group, and the black asterisks indicate the significance between trials in the DCA group. The red asterisks indicate the significance between disease (Healthy and DCA) in Trials 1, 2, and 3.

Figure 5 shows the mean (95% confidence interval) for each parameter in the ballistic targeting task. For ANOVA, Levene’s test was conducted to test for equality variances, and the assumption condition was not met for all parameters (RT: F = 35.803, *p* < 0.01; MV: F = 214.125, *p* < 0.001; MT: F = 103.975, *p* < 0.001; TrajectE: F = 120.858, *p* < 0.001; F = 232.406, *p* < 0.001). Table 1 indicates the results of the Kruskal–Wallis test. Since all parameters showed significant effects for at least one or more of the factors, we performed multiple post hoc comparison tests (Mann–Whitney U test). There were significant differences between the Healthy and DCA groups in each trial for all parameters, except for trial 2 for RT and trial 1 for MV. In the DCA, the RT in trials 2 and 3 was significantly prolonged compared to that in trial 1. In the Healthy group, the MV in trials 1 and 3 was significantly higher than that in trial 2. The MT in trial 3 was shorter than that in trials 1 and 2. TrajectE was significantly smaller in trial 3 than in trial 2.

**Table 1:** The results of the Kruskal–Wallis test.

	H	df	*p*
RT			
Trial	23.758	2	< .001
Disease	2.769	1	0.096
MV			
Trial	7.1	2	0.029
Disease	1.09	1	0.296
MT			
Trial		2	0.023
Disease	75.832	1	< .001
TrajectE			
Trial	6.126	2	0.047
Disease	166.81	1	< .001
targetE			
Trial	1.749	2	0.417
Disease	461.024	1	< .001

RT, Reaction time; MV, Maximum velocity; MT, Movement time; TrajectE, Trajectory error; TargetE, Targeting error.

### Correlation findings

**Figure 6:**
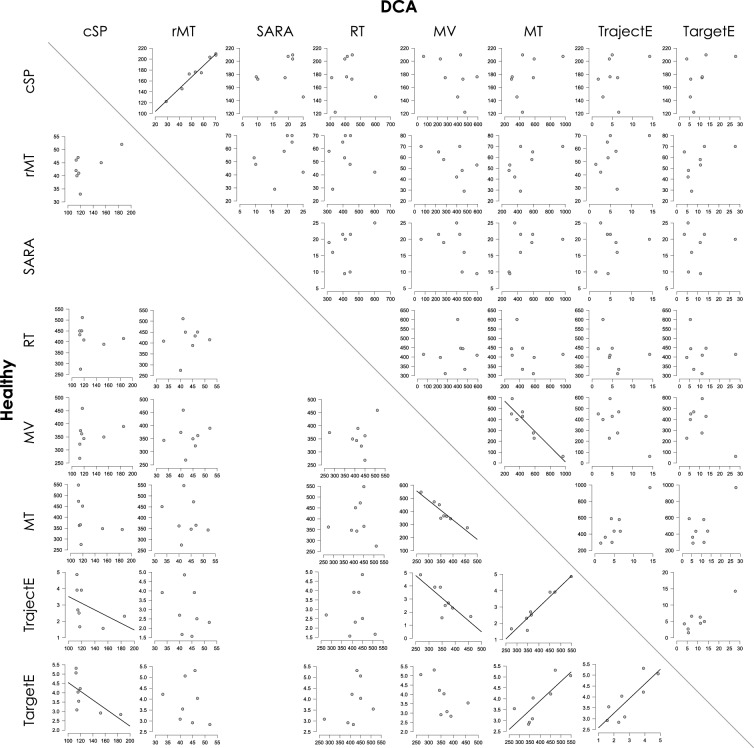
Correlation between cSP and motor impairment index. The scatterplot shows the relationship between each parameter, with the upper notation on the horizontal axis of each graph and the left notation on the vertical axis. The scatter plots are arranged with the lower left side from the diagonal line in the center showing the healthy group and the upper right side showing the degenerative cerebellar ataxia (DCA) group. Graphs showing the regression lines are those in which significant correlations are observed. Abbreviations: cSP, Cortical silent period; rMT, Resting motor threshold; SARA, Scale for assessment and rating of ataxia; RT, Reaction time; MV, Maximum velocity; MT, Movement time; TrajectE, Trajectory error; TargetE, Targeting error.

Table 2 shows the results of the correlation test, and Fig. 6 shows the scatter diagram between cSP, rMT, and the performance indicators of the ballistic targeting task (RT, MV, MT, TrajectE, and TargetE). If the regression line is filled in, it indicates a significant correlation between the two values. cSP-TrajectE, cSP-TargetE, MV-MT, MV-TrajectE, MT-TrajectE, MT-TargetE, and TrajectE-TargetE were detected in the healthy group.

**Table 2:** Results of the correlation test.

Factor A	Fa ctor B	Healthy				DCA			
		SW	*p*	r/rha	*p*	SW	*p*	r/rhc	*p*
cSP	rMT	0.692	0.002	0.19	0.665	0.932	0.53	0.982***	< .001
cSP	SARA					0.95	0.714	0.161	0.703
cSP	RT	0.847	0.09	− 0.073	0.864	0.802	0.03	0.167	0.703
cSP	MV	0.705	0.003	0.543	0.171	0.951	0.725	− 0.499	0.208
cSP	MT	0.686	0.002	− 0.714	0.058	0.848	0.09	0.484	0.224
cSP	TrajeclE	0.763	0.011	− 0.762*	0.037	0.798	0.027	0.238	0.582
cSP	TargatE	0.743	0.007	− 0.762*	0.037	0.812	0.039	0.548	0.171
rMT	SARA					0.951	0.713	0.272	0.514
rMT	RT	0.891	0.239	0.147	0.728	0.84	0.075	− 0.057	0.894
rMT	MV	0.95	0.711	0.074	0.861	0.953	0.739	− 0.565	0.144
rMT	MT	0.965	0.858	− 0.235	0.575	0.849	0.092	0.559	0.149
rMT	TrajeclE	0.968	0.878	− 0.333	0.42	0.814	0.041	0.323	0.435
rMT	TargatE	0.976	0.938	− 0.21	0.617	0.817	0.044	0.539	0.168
SARA	RT					0.861	0.123	0.357	0.385
SARA	MV					0.877	0.176	− 0.544	0.164
SARA	MT					0.826	0.054	0.42	0.301
SARA	TrajeclE					0.825	0.053	0.207	0.623
SARA	TargatE					0.797	0.027	− 0.24	0.568
RT	MV	0.847	0.088	0.161	0.704	0.891	0.239	0.129	0.761
RT	MT	0.866	0.215	− 0.012	0.978	0.833	0.063	− 0.274	0.512
RT	TrajeclE	0.904	0.317	0.017	0.968	0.709	0.003	− 0.476	0.243
RT	TargatE	0.901	0.295	0.384	0.348	0.81	0.036	0.024	0.977
MV	MT	0.916	0.397	− 0.929***	< 0.001	0.855	0.106	− 0.931	< .001
MV	TrajeclE	0.8 55	0.107	− 0.803*	0.016	0.811	0.038	− 0.233	0.582
MV	TargatE	0.864	0.132	− 0.636	0.09	0.698	0.002	0	1
MT	TrajeclE	0.755	0.009	0.905**	0.005	0.821	0.048	0.643	0.096
MT	TargatE	0.945	0.657	0.808*	0.015	0.736	0.006	0.262	0.536
TrajectE	TargatE	0.956	0.772	0.83*	0.011	0.766	0.012	0.69	0.069

In healthy group, tha SARA was nol measured. If significant ol Shapiro–Wilk (SW) last Spearman's test (rfio) was applied. If not significant of SW teal, Pearson's test (r) was applied. SCD, Spinocerebellar ataxia; TrajeclE, Trajectory error; TargetE, Targeting Error; cSP, Cortical silent period; rMT, Resting motor threshold; SARA, Scale For assessment and rating ot ataxia; RT, Reaction time; MV, Maximum velocity; MT, Movement time, *p < .05, **p < 0.01, ***p < 0.001.

## Discussion

This study aimed to investigate the relationship between motor cortex function, specifically that of the GABAergic inhibitory circuits, and motor performance in individuals with DCA. Our findings suggest that the excitability of the GABAergic inhibitory circuit in M1, as estimated by cSP, is significantly prolonged in individuals with DCA compared with that in healthy controls. In the healthy group, cSP correlated with ballistic targeting performance. However, the cSP in DCA did not correlate with the degree of ataxia estimated using SARA or ballistic targeting performance. These findings suggest that, while the excitability of inhibitory circuits in the motor cortex is increased in cases of DCA, this prolongation does not demonstrate a linear correlation with the severity of motor impairment. Furthermore, these findings suggest that the increase in excitability within inhibitory cortical circuits in DCA may not exert the same level of influence on motor control as observed in healthy older adults. However, it is essential to acknowledge the limitations of this study, including the small sample size and the inclusion of only a few subtypes of DCA.

The cSP in DCA was significantly prolonged compared to that in healthy controls. Furthermore, our logistic regression analysis revealed that cSP had significant discriminative power for detecting DCA in healthy populations, indicating that this neurophysiological parameter can be used as a potential biomarker for DCA diagnosis. This finding is consistent with previous studies^12–15^. These studies have reported reduced cerebellar atrophy and inhibition of the contralateral motor cortex (cerebellar inhibition) in individuals with DCA^20,37^. Furthermore, because the cerebellum atrophies over a long period in DCA, other cerebellar areas may undergo functional changes to compensate for this loss of function. For example, it is hypothesized that in motor learning, the ability of error-dependent motor learning involving the cerebellum is reduced and is compensated by use-dependent learning carried out by the motor cortex, thereby delaying the onset of symptoms^38,39^. Motor learning does not occur in a single region but is thought to occur due to the entire brain working as a single system in cooperation with other brain regions. In other words, there may be a compensatory increase in the excitability of inhibitory circuits within the motor cortex due to a decrease in the inhibitory output from the cerebellum to the contralateral side. These findings led to the hypothesis that this excessive prolongation of cSP in the DCA may compensate for the decrease in inhibitory output from the cerebellum to M1 due to cerebellar atrophy. However, there was no correlation between the time since onset and cSP length in this study, and since cerebellar brain inhibition was not measured, further research on this point is necessary.

Factors related to cSP include age^40^ and stimulus intensity^23,34^. Therefore, age-matched healthy participants were selected as the target group in this study. Therefore, this difference in cSP length is unlikely due to age. On the other hand, stimulus intensity had a strong effect on cSP. However, in the present experiment, the intensity was adjusted to 1.3 times the rMT; therefore, the total amount of stimulation to the motor cortex was considered uniform. In addition, there was no significant difference in rMT between the DCA and healthy groups. Based on the above findings, the difference in cSP between patients with DCA and healthy subjects may not be due to rMT or stimulus intensity. Previous studies have reported differences only in cSP, without differences in rMT^41^, which may be mainly due to the involvement of neurotransmitters such as GABA. In contrast, interestingly, a positive correlation was found between cSP and rMT in DCA. cSP and rMT are likely to change independently; therefore, there may be other causes for these changes. This discussion on rMT changes will be updated in future publications based on clinical data.

The cSP is thought to be linked to an individual’s motor control ability. For instance, in motor-stopping tasks, the longer the cSP during 10% MVC, the better the task performance^22^. Additionally, cSP at 100% MVC is shorter than at 10% MVC^23,42^. In other words, when producing strong force, intracortical inhibition is flexibly released, contributing to increased excitability of the corticospinal tract. The maintenance of some level of intracortical inhibition when the subject is near rest, along with its flexible reduction when producing strong force, is thought to contribute to well-controlled output force. Based on these ideas, we hypothesized that cSP may be related to ataxia, a movement disorder affecting force control and coordination. Nevertheless, the relationship between cSP and the severity of ataxia in DCA with abnormalities in cSP remains unclear. In the present study, there was no significant correlation between SARA and cSP, which indicates the severity of ataxia. This may mean that a rough assessment index such as SARA^25^, which includes many other motor skills besides upper limb motor skills, such as walking, standing retention, and lower limb movements, may not be suitable for explaining the pathophysiology of hand ataxia. In other words, the ability of cSP abnormalities to explain changes in SARA, which reflect the severity of whole-body ataxia, may be poor, and cSP derived from hand muscles may reflect hand movement disorders. Therefore, in the present study, we examined performance on a ballistic targeting task to quantitatively capture the characteristics of ataxia: delayed motor onset, prolonged motor time, motor decompensation, and measurement abnormalities.

The involvement of the cerebellum in the performance of the ballistic targeting task has been reported in previous studies^29,43^. In this study, a repetitive visual targeting task involving right wrist movements was performed in both groups. The RT, MV, MT, TrajectE, and TargetE were calculated and compared between healthy controls and patients with DCA. The repetition of this task affected the performance in the RT, MV, MT, and TrajectE, and these parameters in the third trial were significantly different between the Healthy and DCA groups. As mentioned earlier, the cSP reflects force control and the ability to perform the task of stopping the movement^22^. In the present study, the longer the cSP of healthy subjects, the better their performance on the ballistic targeting task. However, there was no correlation between cSP and performance-related measures of the ballistic targeting task in the DCA. A longer cSP was associated with better motor control ability, as indicated by data from previous studies and healthy subjects in the current study. However, in the DCA with longer cSP than in healthy subjects, overall performance was worse and was also found to be unrelated to performance within the DCA. This indicated that a longer cSP in the DCA does not necessarily contribute to motor control.

The ballistic targeting task requires feedforward motor control^44^ based on an internal model^45^ because, with very fast movements, it is difficult to perform only online feedback control^46^. After all, sensory feedback does not arrive in time to correct the movement^44^. Computational theory research has confirmed that feedforward control using an internal model is used in the ballistic motion performed by the human arm^47^. This study also showed that the most efficient motion trajectories are those with unimodal velocity and low jerk, which is consistent with the method of analyzing the trajectory anomalies applied in this study. In other words, TrajectE is considered an effective indicator of the deviation from the optimal trajectory constructed by the internal model. This index and the speed-related parameters improved in the third trial with healthy subjects. This may be because of learning through repetition. The cerebellum is deeply involved in the construction of this internal model, called motor learning, for targeted tasks^45,48^, and such function are impaired in cerebellar patients^49^. However, in this study, individuals with DCA exhibited poor motor performance, and there was no evidence of motor learning similar to that observed in the healthy group. This suggests that the learning ability of individuals with DCA was impaired. In summary, the ballistic reaching motor task differentiated patients with DCA from healthy subjects by showing the characteristics of ataxia due to cerebellar damage: prolonged motor time, motor decomposition, measurement error, and impaired motor learning.

However, an interesting phenomenon was the gradual deterioration of RTs observed in patients with DCA. The extension of RT indicates the prolongation of motor planning^50^, and several reasons could explain this phenomenon. A similar slowdown in repetition has also been observed in language studies^51^, which may reflect the difficulties in adapting to new challenges. Another possible explanation could be that individuals with DCA prioritize accuracy over RT. If the difficulty of the target motion is great, the speed decreases following Fitts' law^52^. This theory is also applicable to the prediction of motor control^53^. Given that the capability for error calibration may be restricted or limited in patients with DCA^54^, they may try to manage error correction by extending the time of motor planning as well as the fully extended movement time. Finally, fatigue may also play a role, as it has been shown to reduce the degree of cerebellar inhibition^55^. Therefore, we hypothesized that repetition might have caused fatigue, which would have altered cerebellar inhibition and decreased performance. Further experiments are required to determine the reasons for these observations.

The present study had several limitations. First, the sample size was relatively small, warranting cautious interpretation of correlation analyses. Increasing the sample size poses challenges, as the number of individuals with DCA with a genetic diagnosis willing to participate in neurophysiologic testing was limited. As a result, clinical studies for DCA often have small sample sizes. For example, in a review of rTMS studies involving patients with cerebellar ataxia, five out of eight studies had a sample size of less than eight^56^. This highlights that clinical studies of rare diseases like DCA often face challenges with limited sample sizes. Consequently, the probability of type II error associated with small sample sizes in statistical analysis should always be carefully considered when interpreting the results. It is important to note that as sample sizes increase in future research, there is a potential for new relationships to be discovered among variables that did not exhibit a significant correlation in this study. Therefore, with larger sample sizes, additional insights and associations may emerge, enhancing our understanding of DCA and its complexities. Second, we did not investigate the relationship between specific types of DCA and neurophysiological parameters measured in this study. Different types of DCA may have different pathophysiological mechanisms, and future studies should investigate this possibility.

## Conclusion

Our findings suggest that the excessive activity of the GABAergic inhibitory circuits of M1 in DCA can be estimated based on cSP. However, this increased inhibition not only fails to contribute to target movement control but also does not show any correlation with SARA, a standard measure of ataxia severity. cSP is an excellent biomarker for distinguishing patients with DCA from normal individuals, but the reason for its prolongation remains unclear. Further research is needed to elucidate changes in motor cortex function in DCA and provide evidence to support effective rehabilitation and medication interventions.

## Data Availability

The datasets generated and analyzed in the current study are available from the corresponding author upon reasonable request.
